# Block sparse Bayes-based fuzzy system for RNA N6-methyladenosine sites prediction

**DOI:** 10.1371/journal.pcbi.1013621

**Published:** 2025-10-30

**Authors:** Leyao Wang, Mengyuan Zhao, Hao Xie, Yuqing Qian, Wenhuan Lu, Yijie Ding, Fei Guo

**Affiliations:** 1 College of Intelligence and Computing, Tianjin University, Tianjin, China; 2 College of Computer Science and Control Engineering, Shenzhen Institutes of Advanced Technology, Chinese Academy of Sciences, Shenzhen, China; 3 School of Computer Science and Engineering, Central South University, Changsha, China; 4 Yangtze Delta Region Institute (Quzhou), University of Electronic Science and Technology of China, Quzhou, China; Georgia Institute of Technology, UNITED STATES OF AMERICA

## Abstract

N6-methyladenosine (m6A) can significantly affect RNA expression, gene regulation, and determination of cell fate. As a common and abundant post-transcriptional modification (PTM) of RNA, m6A is also closely associated with the occurrence of numerous diseases. Thus, identifying the m6A modification site in the RNA sequence is a prerequisite for related research. High-throughput sequencing technology has high requirements and low cost performance. Computational methods have made encouraging progress in site prediction. However, most models only consider the effects of different species, ignoring the simultaneous exploration of RNA modifications in different tissues within the same species. We develop and validate a fuzzy system based on Block Sparse Bayesian Learning (BSBL), named BSBL-TSK-FS, which is a powerful sequence-level m6A prediction model. We introduce a Bayesian method that provides a posterior probability output to produce more sparse solutions so that the model has higher accuracy. The model classifies the m6A sites in several tissues of mouse, human, and rat. Under the five-fold cross-validation method (5-CV), the precision of the BSBL-TSK-FS model is 0.84∼0.95. The accuracy of our model improves by 9.4% over the existing SOTA predictors. BSBL-TSK-FS achieves superior performance over current SOTA methods. Finally, in order to verify the generalizability of the model, we carry out cross-species tests, and the results prove the robustness and adaptability of the model. An accurate and reliable sequence modification prediction model is developed to better understand the complex landscape of methylation modification.

## Author summary

RNA molecules undergo a large number of PTMs that can affect their structure and interaction properties. As the most common type of PTM, N6-methyladenosine (m6A) plays a crucial role in life processes such as gene silencing, cell localization, parental imprinting, and various diseases. Therefore, accurate identification of m6A modification sites from mRNA sequences is of great significance for basic research and drug development. The applicability of experimental methods in large-scale studies is poor. In response to these limitations, computational models have been developed to quickly and economically identify m6A modification sites. In this study, we propose a fuzzy system prediction model, called BSBL-TSK-FS, to identify m6A. We verify the performance of the model on a baseline datasets. Our model, BSBL-TSK-FS, performs well in 11 datasets, with an average AUC value of 0.9619 and an average precision value of 0.9028.

## Introduction

Post-transcriptional modifications (PTM) in RNA are common in all areas of life [1,2]. In addition to regulating RNA life stages, modification sites affect RNA localization, tertiary structure, function, and biogenesis [3,4]. As a result, the biological function of RNA is affected. They are produced by covalent alterations or isomerization of nucleotides, usually involving the addition of chemical groups at different locations in the nitrogenous base or ribose cycle [5–7]. More than 150 PTMs have been identified, of which m6A is the most prevalent type of PTM in RNA [8,9]. As an important epigenetic modification, m6A plays a crucial role in gene silencing, cell localization, parental origin imprinting, and other life processes [10–12]. Regulates RNA localization, transcription, splicing, and stability [13–15]. In addition, it has been linked to diseases such as stomach cancer, obesity, and breast tumors [16–18]. In order to carry out basic research and develop new drugs, it is extremely important to precisely identify the m6A modification sites from mRNA sequences.

Currently, most experimental methods for locating RNA post-transcriptional modifications differ in 3 ways: immunoprecipitation methods, chemical-based detection methods, and enzyme-specific methods. RNA immunoprecipitation dependent methods include MeRIP-Seq [19], m6A-Seq [20], miCLIP [21] and other methods. Pseudo-Seq [22] and AlkAniline-Seq [23] utilize compounds that selectively react with modified ribonucleotides to identify m6A. Specific enzymes are used in methods such as m6A-REF-Seq [24] or DART-Seq [25]. Although these are the current gold standards, they still have certain limitations. For example, experiments require the development of specific protocols for each PTM, sensitivity to cross-reactivity, antibodies, or chemical reactions, and complex protocols can cause bias. They are limited by the availability of compounds or specific antibodies [26,27]. In addition, several methods based on Oxford nanopore technology have been developed, such as Epinano [28], Nanocompore [29], Tombo [30], and CHEUI [31]. These methods are time-consuming, laborious, and expensive. In addition, the slow detection process further limits their applicability to large-scale studies [32,33]. In response to these limitations, computational models have been developed to quickly and economically identify m6A modification sites, making them ideal for large-scale data analysis.

Computational methods have become an attractive option for researchers. iRNA-Methyl is a pioneering predictor specifically designed to identify the m6A site in RNA, using support vector machines combined with hand-extracted features to build a model [34]. m6Apred is a predictor specifically designed to identify m6A sites in the Saccharomyces cerevisiae transcriptome [35]. The predictor is based on physicochemical binary coding and cumulative nucleotide frequency extraction features. SRAMP can be used to predict RNA m6A sites in mammals [36]. It extracts features based on nucleotide binary encoding, secondary structure binary encoding, KSNPF, and KNN score, and assembles three random forests to predict m6A sites. WHISTLE [37] effectively captures key signals associated with m6A modifications by integrating sequence and evolutionary features, and is trained using an SVM. deepSRAMP [38] introduces an isoform-level m6A site prediction framework that leverages BiGRU networks combined with a multi-head attention mechanism to capture complex sequence dependencies. By encoding RNA sequences into fixed-length embeddings, the model effectively extracts deep contextual features and achieves promising predictive performance across multiple tissues and species. These predictors identify m6A modification sites based on specific tissues in a single species. Dao et al. [39] designed iRNA-m6A based on a support vector machine (SVM). Using a one-hot encoding scheme, Liu et al. [40] developed the im6A-TS-CNN tool that predicts using convolutional neural networks (CNNs). TS-m6A-DL is described by Abbas et al. [41] as a method based on deep neural networks (DNNs). A combination of four classification algorithms and three deep learning models is used in the im6APred model presented by Luo et al. [42]. A tool called DL-m6A, which uses three different features encoding schemes, was proposed by Rehman et al. [43]. m6A-TSHub [44] is a comprehensive platform for tissue-specific m6A research, integrating four key modules: m6A-TSDB, m6A-TSFinder, m6A-TSVar, and m6A-CAVar, which support database construction, predictive modeling, and variant impact analysis. It enables systematic exploration of tissue-specific m6A methylation from both low-resolution data and genetic variants across 23 human tissues. Most of these models use one-hot coding, k-mer coding or physicochemical property coding to extract the characterization of RNA sequences. Nevertheless, these methods usually consider only shallow RNA sequence encoding and ignore potential correlations between nucleotides. These models all use tissue-specific datasets, but their accuracy and generality need to be improved.

In this study, we propose a block-sparse Bayesian Learning (BSBL)-based Takagi-Sugeo-Kang fuzzy system (TSK-FS), called BSBL-TSK-FS, to identify m6A. The proposed method is more novel and effective than TSK-FS. In order to achieve complete information extraction, we use the position-specific nucleotide propensity (PSNP) to extract RNA sequence features. Extensive benchmarking experiments were conducted on well-curated datasets, and as a result, BSBL-TSK-FS achieves superior performance than current state-of-the-art methods. Finally, cross-species tests were carried out, and the results obtained prove the robustness and adaptability of the model.

The contributions of this study are summarized as follows.

(1) We improve the TSK fuzzy system on the basis of block sparse Bayesian learning by introducing a Bayesian approach that provides the output of posterior probabilities to produce more sparse solutions. Our model has higher accuracy.

(2) Our model does not require a setting for the penalty factor. The penalty factor in general TSK-FS is a constant to balance the regular and error terms, and the experimental results are very sensitive to this data, and improper settings can cause problems such as overlearning. However, the parameter is automatically assigned in BSBL-TSK-FS.

(3) Compared to traditional task-specific computational tools, our model does not require different coding representations of RNA sequences and can directly predict different types of methylation.

(4) Our model can identify methylation modification sites in various tissues of different species.

The next section displays the model framework, experimental results, sequence analysis, and cross-species validation results, and compares them in detail with other methods. The third part describes the experimental materials and methods, including data set introduction, TSK-FS and BSBL algorithms, feature extraction methods and performance evaluation criteria. Finally the paper is summarized.

## Results

### The BSBL-TSK-FS framework

**Fig 1:**
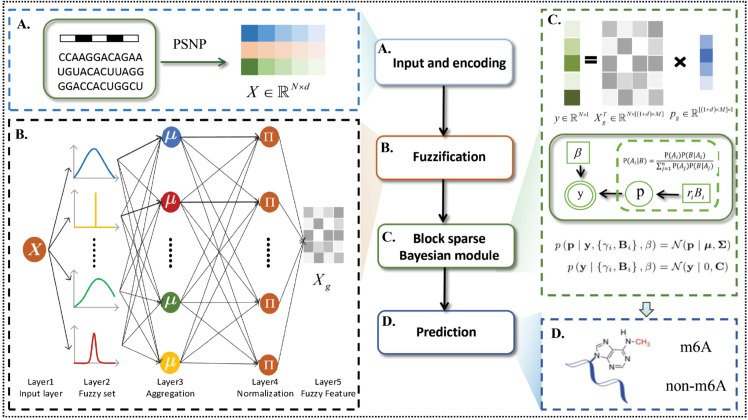
Illustration of the BSBL-TSK-FS model architecture. (A) Input and encoding module. The RNA sequence with length of 41bp was encoded into a matrix via the PSNP with 5-mer. (B) Fuzzification module. The model uses fuzzy system to process the data, then gets fuzzy feature $X_{g}$, and applies $X_{g}$ to the next module. (C) Block sparse Bayesian module. The sparse solution of the model is obtained by block sparse Bayes algorithm, and the parameter **p** is solved. (D) Prediction module. Identify m6A and non-m6A by predicting results.

Our framework uses a block-sparse Bayes-based fuzzy system to predict widely occurring m6A modifications in different tissues of mouse, human, and rat. It consists of four key modules: Input and encoding module, fuzzification module, block sparse Bayes module and prediction module, as shown in Fig 1. The proposed method is mapped to the high dimensional space by fuzzy rules and fuzzy membership function in the fuzzifier [45]. Then we introduce a Bayesian method that provides a posterior probability output to produce more sparse solutions, which improves the accuracy of the model [46,47]. The idea is to find the posterior probability via the Bayesian rule. Given the hyperparameters, the solution is given by the Maximum-A-Posterior estimate [48]. The hyperparameters are estimated from data Maximum Likelihood [49]. More details can be found in the Materials and Methods Section Materials and methods. Our model does not need to set the penalty factor, which is automatically assigned in BSBL-TSK-FS. This avoids problems such as over-learning caused by improper parameter setting. We use 11 widely recognized datasets created by Dao et al. [39], and performe 5-CV on the datasets. These datasets come from different tissues such as brain, heart, kidney, and liver of mouse, humans, and rats. While we applied BSBL-TSK-FS to the task of m6A modification detection, the framework can also be directly applied to other tasks, such as detecting other types modifications.

### BSBL-TSK-FS performance

The main goal of our study was to establish a convenient and reliable predictor that can achieve SOTA accuracy to effectively identify widely occurring m6A modifications from RNA sequences. The Table 1 lists the results of our proposed model on the tissue-specific datasets via five-fold cross-validation (5-CV). Our model, BSBL-TSK-FS, performs well on 11 datasets, with an average AUC value of 0.9619 and an average accuracy value of 0.9028. All other datasets have ACC scores above 84%. On the dataset Rat Liver, this model performed the highest quality, with ACC, MCC and AUC reaching 95.41%, 90.84% and 0.9900, respectively. It also performs well in mouse hearts, with an ACC of 0.9542. Meanwhile, Human Brain’s scores are slightly lower, with ACC, MCC and AUC reaching 84.75%, 69.63% and 0.9231, respectively. AUCs are all above 0.92, and MCCs are all above 0.6963. The mouse brain dataset is the largest and performs poorly, indicating that our model has a slight weakness in handling large data sets. The highest AUC is only 0.07 higher than the lowest AUC. This shows that our model is very stable on the AUC criterion. The BSBL-TSK-FS model demonstrated better performance on small-scale datasets compared to larger ones. As a traditional machine learning method, it tends to be more effective when the sample size is limited. On larger datasets such as Mouse Brain, Human Brain, and Human Kidney, the increased diversity and complexity of the sequences may pose challenges for the fuzzy inference system, which is constrained by the number of fuzzy rules and thus may struggle to capture complex patterns. In addition, the performance of our approach heavily relies on effective feature extraction. The PSTNP module, in particular, has shown higher discriminative power in small datasets, while the greater sequence diversity in large datasets may reduce the effectiveness of the extracted features. These factors may jointly contribute to the slight performance decline observed on large-scale datasets.

**Table 1:** Results of the BSBL-TSK-FS model on m6A datasets under 5-CV.

Species	Tissues	SN	SP	MCC	AUC	ACC
Mouse	Brain	0.8769	0.8183	0.6963	0.9231	0.8475
	Heart	0.9616	0.9468	0.9075	0.9894	0.9542
	Kidney	0.8989	0.8938	0.7928	0.9533	0.8963
	Liver	0.9190	0.8630	0.7833	0.9582	0.8911
	Testis	0.9133	0.8523	0.7669	0.9527	0.8828
Human	Brain	0.8672	0.8485	0.7159	0.9345	0.8578
	Kidney	0.8803	0.8650	0.7452	0.9451	0.8725
	Liver	0.9240	0.9178	0.8421	0.9754	0.9211
Rat	Brain	0.9483	0.9327	0.8810	0.9873	0.9404
	Kidney	0.9311	0.8942	0.8260	0.9716	0.9127
	Liver	0.9560	0.9529	0.9084	0.9900	0.9541

*Note*: MCC, Matthew’s correlation coefficient; SP, specificity; SN, sensitivity; ACC, accuracy; AUC, the area under receiver operating characteristic curve.

**Fig 2:**
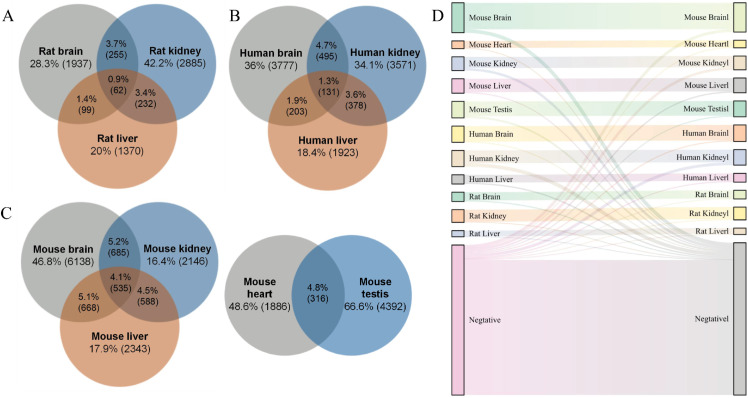
(A–C) Venn diagram of data for different species. (D) Sankey diagram of prediction results for 11 datasets. Straight lines represent correctly classified samples, curved lines represent incorrectly classified samples, and the stronger the lines, the larger the number of samples.

We present the intersections of modifications across tissues in Fig 2A–2C, showing both correlations and significant differences across the tissue data. Some m6A modification sites may occur only in specific tissues, and some may exhibit some similar tendencies in multiple tissues. Therefore, we show the intersection of modifications between tissues. Specifically, we list the exons associated with each modification and treat modifications that share the same exon as intersecting. We find that there are overlaps between the tissues, but the overlaps are less than 5%. Fig 2D is a Sankey chart that visually shows the flow of samples from 11 data sets based on the reality of the prediction label. The curved thin lines represents the misclassified sample. Most of the samples are classified correctly, so they show strong straight lines. It is clear that our method successfully classified the majority of the samples. Our proposed model performs well overall, preliminarily proving its effectiveness in predicting the m6A sites.

### Consensus region analysis

**Fig 3:**
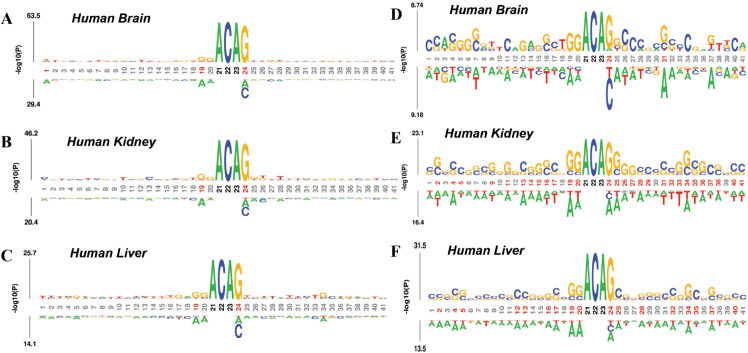
Motif logo analysis on Human datasets. (A–C) Probability Logos of positive samples analysis. (D–E) Probability Logos of positive and negative samples comparative analysis. It’s worth noting that kpLogo uses the “T” to represent the “U” in the RNA sequence.

**Fig 4:**
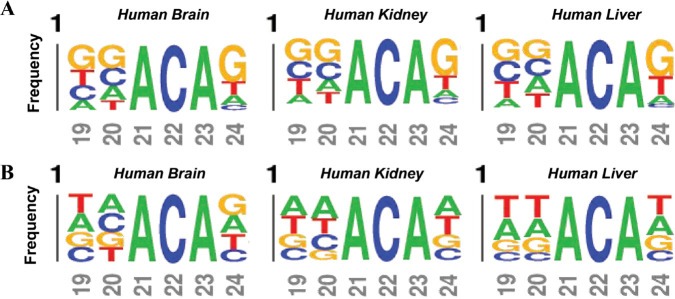
Motif logos in central sequential regions of Human datasets. (A) Motif logos of the positive samples. (B) Motif logos of the negative samples. It’s worth noting that kpLogo uses the “T” to represent the “U” in the RNA sequence.

In order to further understand the mechanism and reason of modification, we use kpLogo [50] to study the distribution of nucleotides around the m6A site. Fig 3A–3C shows the visualization of methylation sequence patterns, we can see that the methylated sequential regions in the tissues are very similar. Fig 3D–3F shows the statistical difference in nucleotide appearance between m6A and non-m6A samples. The top half represents sequences that contain m6A sites, and the bottom half represents sequences that contain non-m6A sites. There are significant differences in the distribution of nucleotides between positive and negative samples (T-test, p value <0.01). The flanking sequences of m6A in all tissues are biased towards GC rich areas, while the flanking sequences of non-m6A are biased towards AU rich areas. It also shows that the idea of constructing m6A classification model by extracting sequence information is reasonable. We present an analysis of the probability distribution of methylation centers in 3 different human tissues. Fig 4A and 4B show the frequency distribution of positive and negative samples of the 3 datasets, respectively. On the one hand, it can be seen that there are significant differences in the motifs of positive and negative samples. On the other hand, it can be seen that the positive sample shows the motif GGACA with the highest frequency in the logos in position from 19 to 23. This is consistent with m6A modifications occurring primarily on the consensus motif DRACH (D-A, G, or U, R-A, or G, H being A, C, or U) [51]. Figs 3 and 4 reveal clear positional differences in nucleotide composition between positive and negative samples, particularly around the central region. These findings support the use of PSKNP, a position-aware encoding scheme that captures local 5-mer preferences across aligned sequences. By leveraging such positional patterns, BSBL-TSK-FS effectively models the sequence context relevant to m6A modifications.

### Ablation analysis

**Fig 5:**
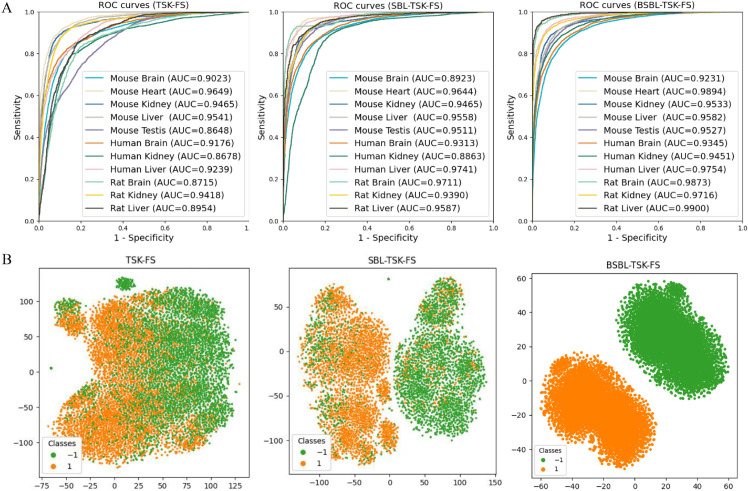
(A) ROC curves of TSK-FS, SBL-TSK-FS and BSBL-TSK-FS models on m6A datasets. (B) Visualization of feature spatial distribution by 3 methods.

To further demonstrate the superiority of BSBL-TSK-FS, we conduct ablation studies. Experiments are conduct on baseline data sets using TSK-FS and SBL-TSK-FS respectively, and the proposed methods are compared in many ways. The comparison results highlight the contribution of sparse Bayesian learning and block sparse Bayesian learning. As shown in Fig 5A, ROC curves of the three methods show that our model has the highest AUC performance. Three species have AUC values above 0.92, 0.93, and 0.97, respectively. In contrast, the TSK-FS method is the worst, while SBL-TSK-FS performs slightly better. The AUC values of the other two methods achieve 0.86-0.97 and 0.89-0.98, respectively. Our method achieves the highest AUC across all datasets, with an average accuracy value of 0.9619, surpassing that of TKS-FS (average AUC 0.9136) and SBL-TSK-FS (average AUC 0.9427). In order to demonstrate the advantages of the proposed method in capturing sequence information, we perform a visual comparison analysis with the two limit methods mentioned above (Fig 5B). We use UMAP to visualize the output features of the three methods. Visualization results show that our approach successfully distinguished the vast majority of negative and positive samples. On the contrary, a small number of negative samples in SBL-TSK-FS are misclassified as positive samples. The worst TSK-FS has a higher incidence of classification errors, resulting in areas of overlap between the two classes.

The results of above 3 methods on Mouse are detailed in Table 2. Our model is superior to the two models in the overall evaluation metrics. Specifically, compared to SBL-TSK-FS method on the Mouse dataset, the accuracy and AUC of our model improve by an average of 1.33% and 0.97%, respectively. TSK-FS perform the worst, especially on the MCC value, with an average of just 0.7138. Compared with TSK-FS, BSBL-TSK-FS improves MCC by 3.5%, 10.33%, 0.53%, 1.25% and 22.18% on these 5 datasets, respectively. TSK-FS performs worst on Mouse Testis, with an ACC value of 0.7721. Our model shows slightly lower SP than TSK-FS on the Mouse Liver dataset. However, its SN is only 0.8828, much lower than that of our model.

**Table 2:** Results of multiple methods on the mouse datasets as analyzed by 5-CV.

Dataset	Tool	MCC	SN(%)	AUC	SP(%)	ACC
Brain	TSK-FS	0.6613	85.41	0.9023	80.43	0.8294
	SBL-TSK-FS	0.6588	86.10	0.8923	79.58	0.8320
	BSBL-TSK-FS	**0.6963**	**87.69**	**0.9231**	**81.83**	**0.8475**
Heart	TSK-FS	0.8042	89.16	0.9649	91.23	0.9020
	SBL-TSK-FS	0.8585	95.43	0.9644	90.22	0.9287
	BSBL-TSK-FS	**0.9075**	**96.16**	**0.9894**	**94.68**	**0.9542**
Kidney	TSK-FS	0.7875	89.47	0.9465	89.29	0.8936
	SBL-TSK-FS	0.7861	89.95	0.9465	88.65	0.8930
	BSBL-TSK-FS	**0.7928**	**89.89**	**0.9533**	**89.38**	**0.8963**
Liver	TSK-FS	0.7708	88.28	0.9541	**88.80**	0.8854
	SBL-TSK-FS	0.7811	91.47	0.9558	86.52	0.8901
	BSBL-TSK-FS	**0.7833**	**91.90**	**0.9582**	86.30	**0.8911**
Testis	TSK-FS	0.5451	79.37	0.8648	75.03	0.7721
	SBL-TSK-FS	0.7612	91.28	0.9511	84.67	0.8798
	BSBL-TSK-FS	**0.7669**	**91.33**	**0.9527**	**85.23**	**0.8828**

The results on Human datasets are detailed in Table 3. The experimental results show that our model is superior to the two models in all evaluation metrics. The performance of TSK-FS is still the lowest, but the MCC is above 0.63, and the performance of SBL-TSK-FS is slightly better, with MCC values between 0.6694 and 0.8349. Compared to SBL-TSK-FS method on the Human dataset, the ACC, MCC and AUC of our model improve by an average of 1.59%, 3.08% and 2.38%, respectively. On these three data sets, our model improves scores of evaluation metrics more significantly. Compared with TSK-FS, BSBL-TSK-FS improves MCC by 3.83%, 10.69% and 16.36% on these 3 datasets, respectively. Moreover, the average ACC and AUC of SBL-TSK-FS are 3.36% and 2.75% higher than that of TSK-FS, respectively.

**Table 3:** Results of multiple methods on the human datasets as analyzed by 5-CV.

Dataset	Tool	MCC	SN(%)	AUC	SP(%)	ACC
Brain	TSK-FS	0.6776	86.63	0.9176	80.92	0.8381
	SBL-TSK-FS	0.7063	86.50	0.9313	84.09	0.8530
	BSBL-TSK-FS	**0.7159**	**86.72**	**0.9345**	**84.85**	**0.8578**
Kidney	TSK-FS	0.6383	80.29	0.8678	83.15	0.8171
	SBL-TSK-FS	0.6694	87.29	0.8863	79.37	0.8332
	BSBL-TSK-FS	**0.7452**	**88.03**	**0.9533**	**86.50**	**0.8725**
Liver	TSK-FS	0.6785	80.75	0.9239	87.03	0.8387
	SBL-TSK-FS	0.8349	92.10	0.9741	91.37	0.9175
	BSBL-TSK-FS	**0.8421**	**92.40**	**0.9754**	**91.78**	**0.9211**

According to Table 4, shows the results of ablation experiments on Rat datasets. Our model generally performs very well, with an average ACC of 0.9357 and an average MCC of 0.8718. The rat is the species that our model predicts most accurately. TSK-FS continues to perform poorly, with the lowest ACC not exceeding 0.8 and the worst MCC only 0.5801. And SBL-TSK-FS performs marginally acceptable, with ACC values between 0.8908 and 0.9298. Compared to SBL-TSK-FS method, the ACC, MCC and AUC of our model improve by an average of 2.26%, 6.36% and 8.01%, respectively. Compared with TSK-FS, the average ACC, MCC and AUC of SBL-TSK-FS improve by 7.83%, 13.15% and 5.34% on these 3 datasets, respectively. Among them, TSK-FS has the highest SP score on Rat Kindey dataset, but the corresponding SN is the lowest. A similar situation is seen with SBL-TSK-FS on the Rat Liver dataset. Overall, our model is more balanced and stable.

**Table 4:** Results of multiple methods on the rat datasets as analyzed by 5-CV.

Dataset	Tool	MCC	SN	AUC	SP	ACC
Brain	TSK-FS	0.5801	90.43	0.8715	66.48	0.7835
	SBL-TSK-FS	0.8584	93.62	0.9711	92.21	0.9298
	BSBL-TSK-FS	**0.8810**	**94.83**	**0.9873**	**93.27**	**0.9404**
Kidney	TSK-FS	0.7538	85.74	0.9418	**89.50**	0.8758
	SBL-TSK-FS	0.7673	92.22	0.9390	83.95	0.8908
	BSBL-TSK-FS	**0.8260**	**93.11**	**0.9716**	89.42	**0.9127**
Liver	TSK-FS	0.6961	85.65	0.8954	83.49	0.8451
	SBL-TSK-FS	0.7989	**96.00**	0.9587	81.94	0.9186
	BSBL-TSK-FS	**0.9084**	95.60	**0.9900**	**95.29**	**0.9541**

### Comparison with other advanced tools

**Fig 6:**
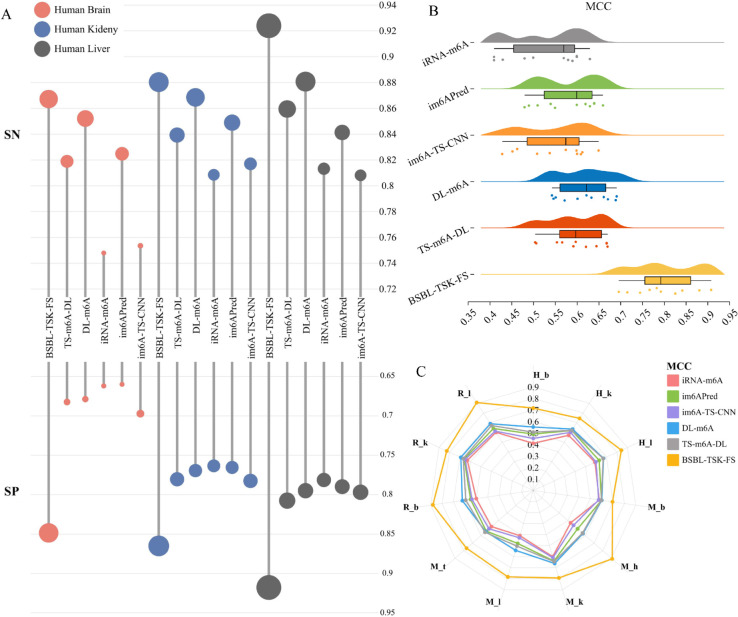
(A) Comparison results between the proposed method and 5 advanced methods on SN and SP indicators. (B) Piano plots of MCC comparisons of six methods across 11 datasets. (C) Radar maps of six methods for MCC comparison on 11 data sets.

**Fig 7:**
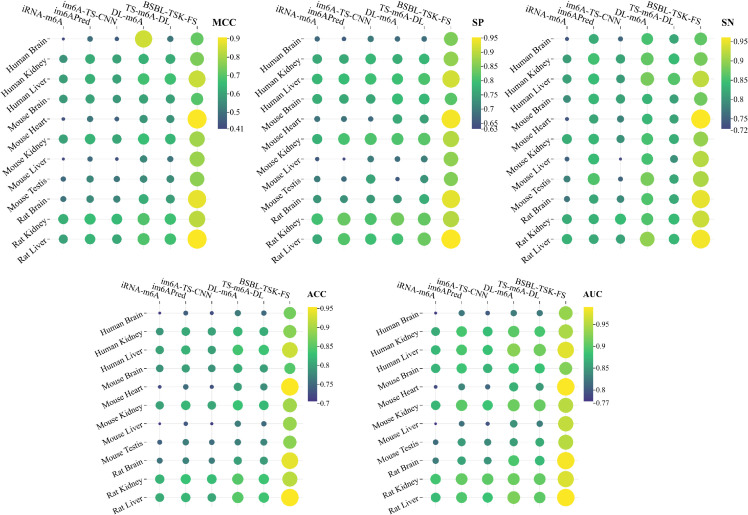
Comparison of six methods on MCC, SN, SP, ACC and AUC indicators. The larger and brighter the bubbles, the higher the value.

To evaluate the effectiveness of our model, we conducted comparisons with several mainstream tools, namely im6A-TS-CNN, im6APred, iRNA-m6A, DL-m6A, TS-m6A-DL, and M6A-BiNP, under a 5-CV framework. The lollipop plot of Fig 6A shows the comparison of six methods SN and SP on three human data sets. It can be seen that our method has the highest performance and has made great progress, especially in SP. Fig 6B and 6C show mcc comparisons across 11 datasets. Fig 6B shows that there is a big gap between other methods and ours in MCC score. It can be intuitively seen that BSBL-TSK-FS is significantly elevated in rat brain, rat liver, and mouse brain (Fig 6C). Fig 7 uses a heat map to show a comparison of ACC, MCC, SN, SP, and AUC scores for different methods on a standard dataset. The brighter the circle, the higher the value. In addition to our approach, DL-m6A and TS-m6A-DL performed better. BSBL-TSK-FS has the best prediction effect on MCC and ACC. The results show that our method is very effective and reliable in m6A modification prediction task.

More detailed comparison results are shown in Tables 5–7. BSBL-TSK-FS has excellent performance on Mouse datasets (Table 5). As shown in the experiment, our proposed model BSBL-TSK-FS shows the best performance compared to SOTA methods on 5 datasets, with an average improvement of 0.99% Acc, 20.44% MCC, and 8.22% AUC compared to other baseline methods. DL-m6A performs slightly better, with an average ACC of 0.7954, an average MCC of 0.585 and an average AUC of 0.8731. BSBL-TSK-FS provides the best accuracy and AUC values on Mouse Heart (0.9542 and 0,9894). Among the methods evaluated, iRNA-m6A, a predictor based on traditional machine learning techniques, showes the lowest overall performance across all 11 datasets, with an Acc of 0.735 and an MCC of 0.47. This observation suggests that deep characterization of RNA sequences has a stronger ability to characterize RNA sequences than shallow characterization. For other methods, TS-m6A-DL and im6A-TS-CNN initialize the RNA sequence mainly based on one-hot encoding, thus ignoring the underlying semantic information.

**Table 5:** Comparison with models on mouse datasets via 5-CV.

Dataset	Tool	MCC	SN(%)	AUC	SP(%)	ACC
Brain	im6A-TS-CNN	0.5749	81.50	0.8705	75.85	0.7867
	im6APred	0.60	83.54	0.8847	76.16	0.7985
	iRNA-m6A	0.58	79.32	0.8701	76.90	0.7875
	DL-m6A	0.6016	83.1	0.8879	79.07	0.8109
	TS-m6A-DL	0.5974	81.34	0.8831	78.36	0.7985
	M6A-BiNP (PSP-PMI)	0.464	74.4	0.818	72.0	0.732
	M6A-BiNP (PSP-PJMI)	0.544	76.8	0.858	77.5	0.772
	BSBL-TSK-FS	**0.6963**	**87.69**	**0.9231**	**81.83**	**0.8475**
Heart	im6A-TS-CNN	0.4633	78.37	0.8115	67.60	0.7299
	im6APred	0.51	81.69	0.8350	68.92	0.7531
	iRNA-m6A	0.44	75.24	0.7948	68.97	0.7276
	DL-m6A	0.5720	83.55	0.8682	76.22	0.7989
	TS-m6A-DL	0.5664	81.50	0.8504	74.96	0.7823
	M6A-BiNP (PSP-PMI)	0.588	80.7	0.880	78.0	0.794
	M6A-BiNP (PSP-PJMI)	0.873	93.7	0.984	93.6	0.937
	BSBL-TSK-FS	**0.9075**	**96.16**	**0.9894**	**94.68**	**0.9542**
Kidney	im6A-TS-CNN	0.6094	79.91	0.8842	81.00	0.8046
	im6APred	0.64	83.51	0.9008	80.42	0.8196
	iRNA-m6A	0.60	82.60	0.8726	77.31	0.7998
	DL-m6A	0.6618	85.21	0.9112	80.98	0.8310
	TS-m6A-DL	0.6451	82.59	0.9079	81.86	0.8222
	M6A-BiNP (PSP-PMI)	0.550	79.5	0.859	75.4	0.775
	M6A-BiNP (PSP-PJMI)	0.696	84.2	0.929	85.3	0.848
	BSBL-TSK-FS	**0.7928**	**89.89**	**0.9533**	**89.38**	**0.8963**
Liver	im6A-TS-CNN	0.4288	72.39	0.7953	70.24	0.7132
	im6APred	0.48	84.01	0.8181	63.05	0.7353
	iRNA-m6A	0.41	74.93	0.7743	65.59	0.7059
	DL-m6A	0.5429	85.08	0.8443	68.31	0.7670
	TS-m6A-DL	0.5044	79.53	0.8288	70.60	0.7506
	M6A-BiNP (PSP-PMI)	0.456	75.5	0.813	70.1	0.728
	M6A-BiNP (PSP-PJMI)	0.702	85.6	0.927	84.5	0.851
	BSBL-TSK-FS	**0.7833**	**91.90**	**0.9582**	**86.30**	**0.8911**
Testis	im6A-TS-CNN	0.5090	75.21	0.8380	75.61	0.7541
	im6APred	0.54	85.70	0.8522	68.09	0.7690
	iRNA-m6A	0.48	78.14	0.8156	70.02	0.7440
	DL-m6A	0.5465	88.21	0.8541	65.62	0.7692
	TS-m6A-DL	0.5544	81.79	0.8630	73.33	0.7756
	M6A-BiNP (PSP-PMI)	0.487	77.7	0.824	70.9	0.743
	M6A-BiNP (PSP-PJMI)	0.701	84.8	0.930	85.2	0.850
	BSBL-TSK-FS	**0.7669**	**91.33**	**0.9527**	**85.23**	**0.8828**

**Table 6:** Comparison with models on human datasets via 5-CV.

Dataset	Tool	MCC	SN(%)	AUC	SP(%)	ACC
Brain	im6A-TS-CNN	0.4523	75.35	0.8029	69.71	0.7253
	im6APred	0.49	82.48	0.8241	65.99	0.7423
	iRNA-m6A	0.41	74.79	0.7756	66.19	0.7126
	DL-m6A	0.8515	85.21	0.8278	67.87	0.7654
	TS-m6A-DL	0.5068	81.91	0.8262	68.23	0.7507
	M6A-BiNP (PSP-PMI)	0.440	71.1	0.793	72.9	0.720
	M6A-BiNP (PSP-PJMI)	0.641	81.0	0.900	83.1	0.820
	BSBL-TSK-FS	0.7159	**86.72**	**0.9345**	**84.85**	**0.8578**
Kidney	im6A-TS-CNN	0.6006	81.70	0.8781	78.25	0.7998
	im6APred	0.62	84.89	0.8896	76.45	0.8067
	iRNA-m6A	0.57	80.85	0.8634	76.34	0.7899
	DL-m6A	0.6335	86.85	0.9009	76.94	0.8190
	TS-m6A-DL	0.6211	83.93	0.8904	78.04	0.8099
	M6A-BiNP (PSP-PMI)	0.493	75.5	0.832	73.8	0.746
	M6A-BiNP (PSP-PJMI)	0.633	80.9	0.896	82.3	0.816
	BSBL-TSK-FS	**0.7452**	**88.03**	**0.9533**	**86.50**	**0.8725**
Liver	im6A-TS-CNN	0.5992	80.81	0.8811	79.69	0.7994
	im6APred	0.63	84.13	0.8915	78.98	0.8155
	iRNA-m6A	0.59	81.32	0.8738	78.13	0.8013
	DL-m6A	0.6714	88.07	0.9259	79.49	0.8378
	TS-m6A-DL	0.6684	85.95	0.9135	80.75	0.8335
	M6A-BiNP (PSP-PMI)	0.550	76.9	0.856	78.1	0.775
	M6A-BiNP (PSP-PJMI)	0.748	87.4	0.951	87.4	0.874
	BSBL-TSK-FS	**0.8421**	**92.40**	**0.9754**	**91.78**	**0.9211**

**Table 7:** Comparison with models on rat datasets via 5-CV.

Dataset	Tool	MCC	SN(%)	AUC	SP(%)	ACC
Brain	im6A-TS-CNN	0.5379	79.04	0.8469	74.23	0.7664
	im6APred	0.55	81.80	0.8580	72.75	0.7727
	iRNA-m6A	0.50	77.00	0.8282	73.47	0.7596
	DL-m6A	0.6221	85.64	0.8930	74.16	0.7990
	TS-m6A-DL	0.5922	82.27	0.8758	76.82	0.7955
	M6A-BiNP (PSP-PMI)	0.570	78.4	0.869	78.6	0.785
	M6A-BiNP (PSP-PJMI)	0.853	91.8	0.980	**93.5**	0.926
	BSBL-TSK-FS	**0.8810**	**94.83**	**0.9873**	93.27	**0.9404**
Kidney	im6A-TS-CNN	0.6500	84.15	0.9017	80.77	0.8246
	im6APred	0.66	82.90	0.9061	83.05	0.8297
	iRNA-m6A	0.63	82.46	0.8877	80.05	0.8178
	DL-m6A	0.6915	85.70	0.9100	83.95	0.8484
	TS-m6A-DL	0.6683	84.00	0.9066	82.81	0.8341
	M6A-BiNP (PSP-PMI)	0.563	79.2	0.868	77.1	0.781
	M6A-BiNP (PSP-PJMI)	0.750	86.78	0.946	88.3	0.875
	BSBL-TSK-FS	**0.8260**	**93.11**	**0.9716**	**89.42**	**0.9127**
Liver	im6A-TS-CNN	0.6126	81.56	0.8830	79.63	0.8059
	im6APred	0.64	82.24	0.8949	81.38	0.8181
	iRNA-m6A	0.60	83.09	0.8766	76.33	0.8090
	DL-m6A	0.6894	89.70	0.9197	80.21	0.8496
	TS-m6A-DL	0.6706	83.47	0.9025	83.48	0.8348
	M6A-BiNP (PSP-PMI)	0.663	82.6	0.912	82.6	0.826
	M6A-BiNP (PSP-PJMI)	0.900	94.6	0.989	**95.4**	0.950
	BSBL-TSK-FS	**0.9084**	**95.60**	**0.9900**	95.29	**0.9541**

On the Human datasets, there is no doubt that ACC and AUC have shown clear signs of improvement (Table 6).The Acc of BSBL-TSK-FS improved by approximately 5.35-9.24% over the next highest predictor (DL-m6A). However, on the Human Brain, our method is slightly lower than DL-m6A in terms of MCC. Our proposed model BSBL-TSK-FS shows the best performance on AUC, with an average improvement of 6.95% compared to other baseline methods. DL-m6A performs slightly better, with an average ACC of 0.8074, an average MCC of 0.7188 and an average AUC of 0.8849. On all three datasets, AUC values for all methods exceed 0.8. BSBL-TSK-FS provides the best accuracy value on Human Liver (0.9211).

On Rat datasets, our model is relatively stable and performs well on all datasets (Table 7). BSBL-TSK-FS provides the best accuracy value on Rat Liver (0.9541). Our proposed model BSBL-TSK-FS shows the best performance on ACC and MCC, with an average improvement of 10.34% ACC and an average improvement of 20.41% MCC, compared to other baseline methods. The AUC alues of our method are higher than DL-m6A method by 0.0943, 0.0616 and 0.0703, respectively. The remaining four methods (iRNA-m6A, im6APred, im6A-TS-CNN and TS-m6A-DL) also achieved reasonable performance on AUC, all above 0.82.

m6A-TSFinder [44] proposed a weakly supervised deep learning framework to predict tissue-specific m6A methylation from low-resolution data and constructed tissue-level models for 23 human tissues. This approach significantly broadened the landscape of m6A prediction beyond base-resolution data. However, due to differences in data resolution, sequence structure, and prediction tasks, our model cannot be directly compared with m6A-TSFinder on the 23 human tissue datasets. To ensure fairness, we evaluated our model on the same benchmark dataset used in the m6A-TSFinder study, enabling direct comparison under an equivalent prediction setting. Detailed performance comparisons are provided in Table 8.

**Table 8:** Performance comparison between different approaches on independent datasets from human tissues.

Tissue	m6A-TSFinder	TS-m6A-DL	im6A-TS-CNN	iRNA-m6A	BSBL-TSK-FS
Brain	0.8132	0.8097	0.8056	0.7845	0.8627
Liver	0.8850	0.8784	0.8805	0.8681	0.9176
Kidney	0.8796	0.8802	0.8727	0.8565	0.8893

*Note*: The results for m6A-TSFinder, TS-m6A-DL, im6A-TS-CNN, and iRNA-m6A in this table are taken from reference [44].

### Performance comparison on the m5C datasets

Our model is capable of predicting not only m6A methylation but also m5C methylation. To assess the performance of the proposed method, we employed the same dataset used by m5C-pred [52]. Table 9 summarizes the comparison results. On dataset *M*.*musculus*, BSBL-TSK-FS achieves the best ACC value (0.9088) with improvements of 14.86% and over the m5C-pred. On dataset *A*.*thaliana*, the accuracy and MCC of our prediction are also significantly improved.

**Table 9:** Performance comparison on the m5C datasets.

Species	Tool	SN(%)	SP(%)	MCC	ACC
*A*.*thaliana*	m5C-pred	67.49	77.42	0.4514	0.7245
	BSBL-TSK-FS	**80.58**	**88.76**	**0.6958**	**0.8467**
*M*.*musculus*	m5C-pred	76.59	75.40	0.5201	0.7602
	BSBL-TSK-FS	**90.30**	**91.48**	**0.8177**	**0.9088**

### Cross-species and cross-tissues prediction analysis

**Fig 8:**
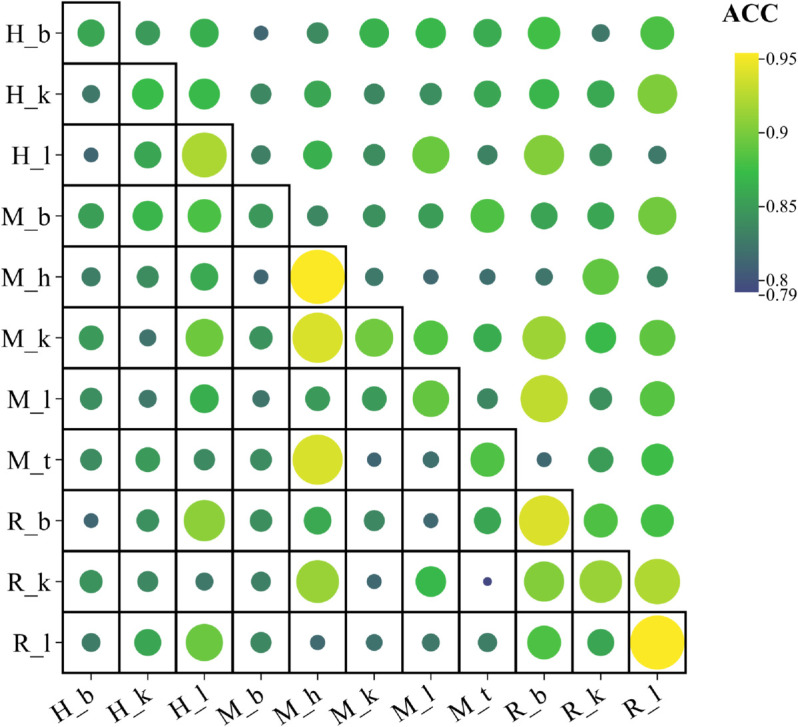
Heap map showing ACCs of cross-species and cross-tissues prediction accuracies.

We conducte cross species and cross tissue experiments to demonstrate that the predictor is not dependent on species and tissue. The result is shown in Fig 8, where each circle in the heat map represents the accuracy obtained. The rows represent training sets and the columns represent test sets. The accuracy of cross-tissue prediction may be affected by factors such as differences in sample size, intra-dataset redundancy, and random noise. All prediction accuracy is higher than 0.79. It can be seen that using different species or tissues for prediction can also achieve good accuracy.

## Discussion

In this paper, we designed a BSBL-TFS-FS model to detect RNA m6A sites in a variety of tissues. We apply fuzzy systems to block sparse Bayesian learning. Compared with the traditional fuzzy network, this method has better approximation performance. We tested the benchmark dataset. The experimental results show that BSBL-TFS-FS is an effective model for sequence prediction. Compared with existing predictors, BSBL-TFS-FS has higher predictive performance. Our model can predict methylation sites in different tissues of different species. To account for tissue- and species-specific characteristics of m6A modifications, we trained separate models for each tissue type and organism. This design ensured that each model was optimally adapted to its respective dataset. Moreover, while our framework is not fully interpretable in the strictest sense, it provides greater interpretability compared to deep learning-based methods. This is mainly due to its rule-based fuzzy inference system and structured Bayesian framework, which together offer clearer insight into the relationship between input features and prediction outcomes. Next, we want to change the form of converting sequences into numerical values through physicochemical properties and then input into the model, and instead use biological sequences directly as inputs to the model.

## Materials and methods

### Benchmark datasets

Zhang et al. [24] developed a technique for detecting m6A sites in different tissues. Based on this study, Dao et al. [39] constructed high-quality benchmark datasets for computational methods. Each dataset contains 41nt long sequences of m6A and non-m6A sites. Using CD-HIT, a sequence similarity score of less than 80% was achieved. Table 10 shows the summary of tissue-specific datasets. Table 11 presents information on independent datasets from three human tissues.

**Table 10:** Summary of tissue-specific m6A datasets.

Species	Tissues	Positive	Negative
Mouse	Brain	8025	8025
	Heart	2201	2201
	Kidney	3953	3953
	Liver	4133	4133
	Testis	4707	4707
Human	Brain	4605	4605
	Kidney	4574	4574
	Liver	2634	2634
Rat	Brain	2352	2352
	Kidney	3433	3433
	Liver	1762	1762

**Table 11:** Summary of m6A independent datasets from human tissues.

Species	Tissues	Positive	Negative
Human	Brain	4604	4604
	Kidney	4573	4573
	Liver	2634	2634

This study also utilized high-quality m5C datasets for *Musmusculus* and *Arabidopsisthaliana* from the work of Abbas et al. [52], which were retrieved from the GEO database (accession numbers GSE93751 and GSE94065) and processed using CD-HIT to remove redundant sequences with more than 70% similarity. Detailed information is provided in Table 12.

**Table 12:** Summary of m5C datasets.

Species	Positive	Negative
*M*.*musculus*	4563	4563
*A*.*thaliana*	5289	5289

### Position-specific nucleotide propensity

In bioinformatics, the position-specific nucleotide propensity (PSNP) have become a popular method for predicting the sites of biological sequences [27,32]. PSNP is an approach that extracts information from sequences by computing the frequency of nucleotides at certain positions. Most mammalian m6A sequences are found within the consensus motif DRACH [51,53]. Therefore, we use 5-mer nucleotides to calculate the frequency and get 4^5^ combinations. For a sequence of length 41nt, we get a 37-dimensional vector.

### BSBL-TSK-FS

#### Fuzzy system.

Given *N* samples $𝐗={\left[{𝐱}_{1},\ldots ,{𝐱}_{i},\ldots ,{𝐱}_{N}\right]}^{T}\in R^{N\times d}$, where ${𝐱}_{i}=\left[x_{i1},x_{i2},\ldots ,x_{id}\right]\in R^{1\times d}$. We notate the label vector in the form of $𝐲\in R^{N\times 1}$. Suppose the 1-order TSK fuzzy system has *K* fuzzy rules, then the *k*-th rule as follows [54,55].

$$\text{if }x_{i1}\text{ is }A_{1}^{k}\wedge x_{i2}\text{ is }A_{2}^{k}\wedge \ldots \wedge x_{id}\text{ is }A_{d}^{k},\;\text{then }f^{k}\left({𝐱}_{i}\right)=p_{0}^{k}+p_{1}^{k}x_{i1}+\ldots +p_{d}^{k}x_{id},k=1,2,\ldots ,K,$$ (1)

where ∧ represents a fuzzy conjunction operator and $A_{j}^{k}$ refers to the fuzzy subset. Input vector ${𝐱}_{i}$ corresponds to each rule, which maps the fuzzy set *A^k^* in the input space to the fuzzy set $f^{k}\left({𝐱}_{i}\right)$ in the output space. The Gaussian membership function is applied to the sample in the if-parts.

$$\mu_{A_{j}^{k}}\left(x_{ij}\right)=\exp\left(\frac{-\left(x_{ij}-c_{j}^{k}\right)}{2\delta_{j}^{k}}\right).$$ (2)

Fuzzy C-means (FCM) clustering [56–58] can be used to determine the membership function mean $c_{j}^{k}$ and variance $\delta_{j}^{k}$.

$$c_{j}^{k}=\frac{\sum_{i=1}^{N}\mu_{ik}x_{ij}}{\sum_{i=1}^{N}\mu_{ik}},$$ (3)

$$\delta_{j}^{k}=\frac{h\sum_{i=1}^{N}\mu_{ik}{\left(x_{ij}-c_{j}^{k}\right)}^{2}}{\sum_{i=1}^{N}\mu_{ik}},$$ (4)

where *h* is a manually adjustable coefficient, and $\mu_{ik}$ represents the fuzzy membership of the *i*-th sample within the *k*-th cluster.

The fuzzy membership function $\mu^{k}({𝐱}_{i})$ and normalized fuzzy membership $\tilde{\mu }({𝐱}_{i})$ for fuzzy set *A^k^* are defined as

$$\mu^{k}({𝐱}_{i})=\prod_{j=1}^{d}\mu_{A_{j}^{k}}\left(x_{ij}\right),$$ (5)

$${\tilde{\mu }}^{k}({𝐱}_{i})=\frac{\mu^{k}({𝐱}_{i})}{\sum_{k^{\prime }=1}^{K}\mu^{k^{\prime }}({𝐱}_{i})}.$$ (6)

The output is given by

$$y^{o}\left({𝐱}_{i}\right)=\sum_{k=1}^{K}\tilde{\mu }\left({𝐱}_{i}\right)f^{k}\left({𝐱}_{i}\right).$$ (7)

When the if-part of the parameter is determined, then ${\tilde{\mu }}^{k}\left({𝐱}_{i}\right)$ is determined. Let

$${𝐱}_{e}=\left(1,{𝐱}_{i}\right)\in {𝐑}^{1\times (1+d)},$$ (8)

$${\tilde{𝐱}}_{i}^{k}={\tilde{\mu }}^{k}\left({𝐱}_{i}\right){𝐱}_{e}\in {𝐑}^{1\times (1+d)},$$ (9)

$${𝐱}_{gi}=\left({\tilde{𝐱}}_{i}^{1},\ldots ,{\tilde{𝐱}}_{i}^{k},\ldots ,{\tilde{𝐱}}_{i}^{K}\right)\in {𝐑}^{1\times [(1+d)\times K]}.$$ (10)

A set of parameters for the then-parts can be expressed as

$$𝐩={\left({\left({𝐩}^{1}\right)}^{T},\ldots ,{\left({𝐩}^{k}\right)}^{T},\ldots ,{\left({𝐩}^{K}\right)}^{T}\right)}^{T},$$ (11)

where ${𝐩}^{k}=((p_{0}^{k}{)}^{T},(p_{1}^{k}{)}^{T},\ldots ,(p_{d}^{k}{)}^{T}{)}^{T}\in {𝐑}^{(1+d)\times 1}$ and $𝐩\in {𝐑}^{[(1+d)\times K]c1}$.

Therefor, the output can be rewritten as

$$y^{o}\left({𝐱}_{i}\right)={𝐱}_{gi}𝐩.$$ (12)

#### Block sparse Bayesian learning.

Let ${𝐗}_{g}=\left[{𝐱}_{g1};{𝐱}_{g2};\ldots ;{𝐱}_{gN}\right]\in R^{N\times [(1+d)\times K]}$. Then we can obtain

$$y={𝐗}_{g}𝐩.$$ (13)

First, we assume that the data is divided into i=1,⋯,M blocks and all the sources ${𝐩}_{i}(\forall i)$ are mutually independent, and the density of each ${𝐩}_{i}$ is Gaussian, given by

$$p\left({𝐩}_{i};\gamma_{i},{𝐁}_{i}\right)∼𝒩\left(0,\gamma_{i}{𝐁}_{i}\right),\;i=1,\cdots ,M$$ (14)

where $\gamma_{i}$ is a nonnegative hyperparameter controlling the row sparsity of ${𝐩}_{i}$, representing the correlation of the *i*th block of data. For smaller $\gamma_{i}$, the correlation ${𝐩}_{i}$ is noise. When $\gamma_{i}=0$, the associated ${𝐩}_{i}$ becomes 0. $\gamma_{i}{𝐁}_{i}$ is the covariance matrix of ${𝐩}_{i}$. ${𝐁}_{i}$ is a positive-definite matrix that captures the structure of the correlation of ${𝐩}_{i}$ that needs to be estimated.

Further, assuming that blocks are uncorrelated with each other, it can be modeled as

$$p\left({𝐩}_{i}|\left\{\gamma_{i},{𝐁}_{i}\right\}\right)=𝒩(𝐱;0,\Gamma ),$$ (15)

where

$$\Gamma =\left[\begin{array}{ccc} \gamma_{1}{𝐁}_{1} & & \\ & \ddots & \\ & & \gamma_{M}{𝐁}_{M} \end{array}\right].$$ (16)

For the observation vector *y*, it is assumed to obey the following probability density distribution

$$p(𝐲|\beta )=𝒩\left(𝐲;{𝐗}_{g}𝐩,\beta^{-1}𝐈\right).$$ (17)

The posterior probability density and the likelihood function can be obtained by utilizing Gauss’s constant equation.

$$p\left(𝐩|𝐲,\left\{\gamma_{i},{𝐁}_{i}\right\},\beta \right)=𝒩(𝐩|\mu ,\Sigma )\;p\left(𝐲|\left\{\gamma_{i},{𝐁}_{i}\right\},\beta \right)=𝒩(𝐲|0,𝐂)$$ (18)

where

$$\Sigma^{-1}=\Gamma^{-1}+{𝐗}_{g}^{T}\beta {𝐗}_{g}\;\mu =\Sigma {𝐗}_{g}^{T}\beta 𝐲\;𝐂=\beta^{-1}𝐈+{𝐗}_{g}\Gamma {𝐗}_{g}^{T}$$ (19)

In order to estimate the covariates ${𝐁}_{i}$, a two-type maximum likelihood approach can be used to obtain the cost function ℒ:

$$ℒ\left(\left\{\gamma_{i},{𝐁}_{i}\right\},\beta \right)≜-2\log p\left(𝐲|\left\{\gamma_{i},{𝐁}_{i}\right\},\beta \right)\;=\log|𝐂|+{𝐲}^{T}{𝐂}^{-1}𝐲.$$ (20)

#### Estimation of hyperparameters.

Using a matrix to find the inverse equation, Eq (20) can be written as

$$ℒ=\left(\sum_{i=1}^{g}\log\left|\gamma_{i}{𝐁}_{i}\right|-N\log|\beta |+\log\left|\Sigma^{-1}\right|\right)\;+\left(\beta \|𝐲-{𝐗}_{g}\mu {\|}_{2}^{2}+\mu^{T}\Gamma^{-1}\mu \right).$$ (21)

Taking partial derivatives separately yields

$$\frac{\partial ℒ}{\partial \gamma_{i}}=\frac{d_{i}}{\gamma_{i}}-\mathrm{Tr}\left[\frac{{𝐁}_{i}^{-1}\left(\mu_{i}\mu_{i}^{T}+\Sigma_{i}\right)}{\gamma_{i}^{2}}\right],\;\forall i\;\frac{\partial ℒ}{\partial {𝐁}_{i}}={𝐁}_{i}^{-1}-\gamma_{i}^{-1}{𝐁}_{i}^{-1}\left[\Sigma_{i}+\mu_{i}\mu_{i}^{T}\right]{𝐁}_{i}^{-1},\;\forall i\;\frac{\partial ℒ}{\partial \beta }=-\frac{N}{\beta }+\mathrm{Tr}\left[\Sigma {𝐗}_{g}^{T}{𝐗}_{g}\right]+\|𝐲-{𝐗}_{g}\mu {\|}_{2}^{2}.$$ (22)

Furthermore, the updated formula can be obtained

$$\gamma_{i}=\frac{1}{d_{i}}\mathrm{Tr}\left[{𝐁}_{i}^{-1}\left(\Sigma_{i}+\mu_{i}\mu_{i}^{T}\right)\right],\;\forall i,\;{𝐁}_{i}=\frac{\left[\Sigma_{i}+\mu_{i}\mu_{i}^{T}\right]}{\gamma_{i}},\;\forall i\;\beta =\frac{N}{\|𝐲-{𝐗}_{g}\mu {\|}^{2}+\mathrm{Tr}\left[\Sigma {𝐗}_{g}^{T}{𝐗}_{g}\right]}.$$ (23)

Algorithm 1 describes the entire process of BSBL-TSK-FS, and Fig 1 shows the framework of the proposed approach.

**Algorithm 1 Algorithm of BSBL-TSK-FS model.**

**Require:** The training set $𝐗\in R^{N\times d}$, number of blocks *M*, number of fuzzy rules *K*, adjustable parameter *h*;

**Ensure:** The prediction labels $𝐲\in R^{N\times 1}$;

1: Determine the mean $c_{j}^{k}$ and variance $\delta_{j}^{k}$ using the FCM method;

2: Calculate the normalized fuzzy membership ${\tilde{\mu }}^{m}\left({𝐱}_{t}\right)$ by Eq (6);

3: Construct the dataset ${𝐗}_{g}=\left[{𝐱}_{g1};\ldots ;{𝐱}_{gi};\ldots ,{𝐱}_{gN}\right]\in R^{N\times [(1+d)\times K]}$ using the fuzzy rules mapped to the new feature space, where ${𝐱}_{gt}$ are obtained by Eq (10);

4: Initialize *γ* and set γ=1;

5: Initialize *β* and set $\beta =1e^{-2}\|𝐲{\|}_{2}$;

6: **while** $\frac{\left\|\gamma^{new}-\gamma \right\|}{\left\|\gamma \right\|}>\eta$ **do**

7:   Calculate Σ,μ by Eq (19);

8:   Calculate *β*, $\gamma_{i},\forall i$ and intra-block correlation coefficient *r* by Eq (23);

9:   Calculate ${𝐁}_{i},\forall i$ by Eq (23);

10:   Calculate *C* by Eq (19);

11: **end while**

12: Estimate sparse solution 𝐩=μ, parameter estimation $\left\{\gamma_{i}{𝐁}_{i}\right\}$ *β*.

13: Estimate **Y** by Eq (13).

### Evaluation metrics

Model evaluation of methylation datasets is based on Matthews correlation coefficient (MCC), specificity (SP), accuracy (ACC) and sensitivity (SN) [59–61]. Moreover, our model was evaluated objectively by calculating the AUC [36]. There is a range of AUC values between 0 and 1. Generally, models with higher AUCs perform better.
